# (-)-Sativan Inhibits Tumor Development and Regulates miR-200c/PD-L1 in Triple Negative Breast Cancer Cells

**DOI:** 10.3389/fphar.2020.00251

**Published:** 2020-03-13

**Authors:** Fu Peng, Liang Xiong, Cheng Peng

**Affiliations:** ^1^ Key Laboratory of Drug-Targeting and Drug Delivery System of the Education Ministry and Sichuan Province, West China School of Pharmacy, Sichuan University, Chengdu, China; ^2^ Key Laboratory of Systematic Research of Distinctive Chinese Medicine Resources in Southwest China, Chengdu University of Traditional Chinese Medicine, Chengdu, China

**Keywords:** (-)-sativan, epithelial-to-mesenchymal transition, triple negative breast cancer, miR-200c, PD-L1

## Abstract

Epithelial-to-mesenchymal transition (EMT) in cancer cells could convert epithelial-like cells to mesenchymal-like cells, resulting in the increased capacity of migration and invasion of cancer cells, and is an essential step in triple negative breast cancer (TNBC) development. Recent reports exert that these EMT-activated TNBC cells are more resistant to immune attacks, with high levels of programmed death ligand1 (PD-L1). Hence, it is worthwhile to find an effective approach in inhibiting EMT-activated TNBC cells. (-)-Sativan (SA) is a naturally isolated isoflavane and could be isolated from *Spatholobus suberectus*, a common traditional Chinese medicine used for breast cancer treatment. It was the first time that SA exerted anti-cancer effects on breast cancer cells, according to our study. In this study, SA displayed a significant inhibitory effect on the proliferation of TNBC cells by inducing apoptosis. SA increased Bax expression, and decreased Bcl-2 protein levels. SA inhibited cell migration and invasion of MDA-MB-231 and BT-549 cells. SA could decrease N-cadherin, Snail, Vimentin, and PD-L1 expression. SA increased miR-200c expression, and decreased PD-L1 expression. Luciferase assay showed that miR-200c directly targeted PD-L1. SA promoted tumor cell susceptibility to CTL-mediated lysis. Further study confirmed that SA could inhibit PD-L1 expression and EMT by up-regulating miR-200c. *In vivo* results displayed that SA could also inhibit tumor volumes and weights. These findings indicate that SA exerts an inhibitory effect on TNBC cell proliferation, migration, invasion, and tumor gtrowth, and partly provide evidence for the anti-breast cancer effect of *Spatholobus suberectus* Dunn in TNBC therapy.

## Introduction

Breast cancer, specifically triple negative breast cancer (TNBC), is ranked as the leading cause of death among women worldwide (Siegel et al., 2013). Clinical data suggested that TNBC tended to occur in younger women (Carey et al., 2006). The widely used breast cancer treatments include surgery, radiation, hormone therapy, chemotherapy, and targeted therapy. However, about 10% to 20% of breast cancer patients suffering from TNBC did not respond to the standard therapy well (Gralow et al., 2008). Thus, it is imperative to search for a novel anti-TNBC approach with novel molecular targets.

Epithelial-to-mesenchymal transition (EMT) in cancer cells could convert epithelial-like cells to mesenchymal-like cells, resulting in the increased capacity of migration, invasion, and even drug resistance of cancer cells. Mesenchymal-epithelial transition (MET) is the opposite process of EMT, promoting stabilization of metastatic tumors (Demirkan, 2013). A recent study exerted that EMT contributed a critical step in the progression of TNBC (Drasin et al., 2011). Programmed cell death ligand 1 (PD-L1) expressed in cancer cells is a functional ligand of programmed cell death 1 (PD-1) expressed in immune cells (Okazaki and Honjo, 2007), and high expression of PD-L1 could affect the evasion of host antitumor immunity of cancer cells, potentially abating the efficacy of anticancer therapies (Hino et al., 2010; Mittal et al., 2014). Additionally, high expression of PD-L1 was detected in TNBC clinical cases (Dill et al., 2017; Barrett et al., 2018). A recent study indicated that mesenchymal tumors with increased PD-L1 expression (Mak et al., 2016), promoted cell proliferation (Li et al., 2017). Thus, it would be of major interest to find an anti-TNBC drug candidate inhibiting EMT process and PD-L1 expression.

Since 2005, microRNAs (miRNAs) gradually began to be recognized as important breast cancer hallmarks, due to their effects on cell proliferation, immune disability, and metastatic acquisition (Iorio et al., 2005). Targeting specific miRNAs *via* a single compound from Traditional Chinese medicine (TCM) has become a promising approach in breast cancer treatment (Qian et al., 2013; Momtazi et al., 2016; Peng et al., 2019a). It is of interest to identify agents regulating miRNA, capable of modulating EMT and PD-L1.

**Figure 1:**
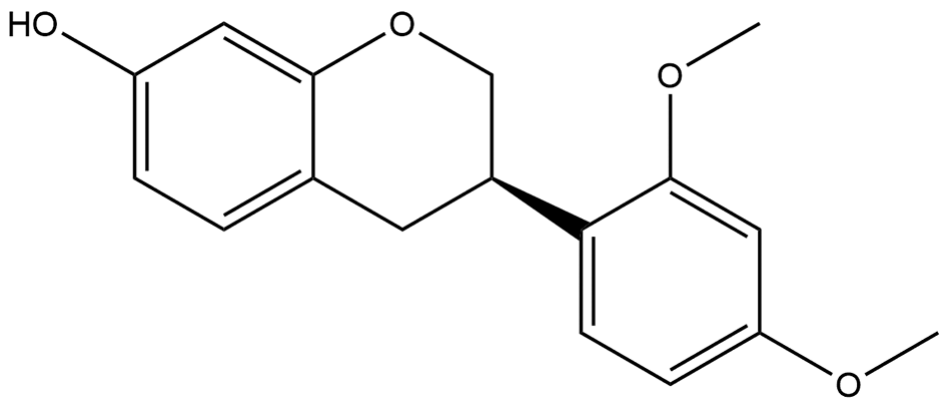
The chemical structure of SA constructed using ChemBioDraw.

*Spatholobus suberectus* Dunn is a traditional Chinese herb commonly used in China for treating blood-stasis related diseases such as breast cancer. *Spatholobus suberectus* Dunn is also traditionally used for the treatment of anemia, menoxenia, and rheumatism (Huang et al., 2013). This herb has been reported to have anti-inflammatory, antioxidant, and antirheumatic effects (Ha et al., 2013). Nowadays, some traditional Chinese medicine physicians tend to utilize *Spatholobus suberectus* Dunn to treat breast cancer patients, and report good responses. Recent studies stated that *Spatholobus Suberectus* possessed potent anti-cancer effects on breast cancer with the capacity of triggering apoptosis, arresting cell cycle and inhibiting lactate dehydrogenase (Wang et al., 2013). Also, *Spatholobus suberectus* Dunn exerted an inhibitory effect on breast cancer migration through the MAPK PI3K/AKT pathway. (Sun et al., 2016). (-)-Sativan (SA) (**Figure 1**) is a naturally isolated isoflavane and could be isolated from *Spatholobus suberectus* Dunn. according to our previous study (Peng et al., 2019b). SA is also exerted in *Lotus corniculatus* Linn, *Trifolium campestre* Schreb., and *Trifolium dubium* Sibth. (Bonde et al., 1973; Ingham, 1978). Subsequently, a report in 2010 exerted a novel synthetic access to SA (Takashima et al., 2010). In this study, it was the first time the anti-breast cancer effect of SA on TNBC cells was reported. SA could inhibit TNBC cell proliferation, migration, invasion, and tumor growth. Additionally, SA exerted an inhibitory effect on EMT process and PD-L1 expression. We also found that SA could up-regulate miR-200c and demonstrated that PD-L1 was a downstream target of miR-200.

## Materials and Methods

### Chemicals and Reagents

All reagents applied in this study were purchased from standard companies with the demanding requirement. Specifically, SA had a 98% purity and was obtained from Chem Faces (Wuhan, CN), and Medkoo (Morrisville, USA). The stocking solution of SA dissolved in DMSO would be stored in -20°C, at most for one month. For the usage of SA, the percentage of DMSO in treatment medium is less than 0.1%. Xylene, Eosin Y, Hematoxylin, and other commonly used chemicals were prepared from Sigma (St. Louis, MO). Primary and secondary antibodies were mainly obtained from Cell Signaling Technology (Danvers, MA). ECL Advance reagent was purchased from Merckmillipore (St. Louis, MO). RNAiso Plus reagent and PrimeScript RT Reagent Kit with gDNA Eraser were obtained from TaKaRa (Bio Inc., Shiga, JP). ExiLENT SYBR Green master mix was obtained from Exiqon (Vedbaek, DK).

### Cell Culture

MDA-MB-231, BT549, and MCF-7 cells were incubated in the 5% CO_2_ 37°C incubator, from American Type Culture Collection (ATCC, USA). CTLL-2 and 293T cells were also obtained from ATCC. All the mediums, FBS, and penicillin were obtained from Gibco (Life Technologies, USA). All the medium was added with 10% FBS, 1% penicillin, and 1% streptomycin. MDA-MB-231, BT-549, and 293T cells were cultured in DMEM medium, while MCF-7 and CTLL-2 cells were cultured in RPMI 1640 medium.

### CCK-8 Assay

MDA-MB-231 and BT549 cells were seeded in the 96 well plates at a density of 5×10^3^ cells/well. After exposure to SA at 24h and 48h, the cell viability was detected through CCK-8 kit, obtained from MedChemExpress (USA), according to the instruction in the kit. After SA interference, all the cells were treated with 10μl reagent for 2h at 37°C in the 5% CO_2_ incubator. Then, the absorbance of the reacted reagent would be read by an ELISA plate reader at 450nm. The experiments were repeated at least three times.

### Flow Cytometry Analysis

The apoptosis analysis kit, equipment, and software were provided by BD Company (CA, USA). The whole experiment was conducted according to the instructions from the manufacturer. Briefly, we seeded MDA-MB-231 and BT549 cells in the 6-well plates at a density of 5×10^5^ cells/well for 24h treatment. After expose to SA, the harvested cells were diluted into 1X Binding Buffer, reaching at 1 × 10^6^ cells/ml. Then, the diluted cells were treated with FITC-conjugated Annexin V and PI for 15min at room temperature at dark. Stained samples needed to be accessed by FACSAria SORP. The results were evaluated according to the cell numbers by FlowJo Software. The independent experiments would be repeated triply.

### Immunoblotting

Cell lysis buffer from Sigma (St. Louis, MO) was used for the whole protein extraction. 20μg protein from MDA-MB-231, BT549, and MCF-7 cells was resolved on 10% SDS-PAGE gels, and transferred onto PVDF membrane from Merckmillipore (St. Louis, MO). After blocking with 5% BSA, sliced membranes with primary antibodies against Bax, Bcl-2, E-cadherin, N-cadherin, Vimentin, Snail, Slug, β-catenin, PD-L1, and β-actin were incubated at 4°C overnight. β-actin was selected as the loading control in western blot. The cut membranes were washed with TBST three times for 5min each wash. After the probing with a secondary antibody, the observations were conducted by ECL Advance reagent.

### Wound Scratch Assay

3×10^5^ cells/ml of MDA-MB-231 and BT-549 cells were plated into Culture-Insert (ibidi GmbH, Martinsried, DE) according to the instruction from the manufacturer. After the cell attachment, Culture-Insert was gently removed, and cells were treated with SA for 24h. Images were captured at the beginning of the experiment and at the end of the experiment. The results were recorded photographically through Zeiss microscope Axio Lab A1. Triplicate experiments were performed independently.

### Chamber Migration and Invasion Assays

Chambers for detecting the migratory and invasive ability of MDA-MB-231 and BT549 cells were obtained from Corning Inc (Coring, USA). The chambers for invasion assay were coated with Matrigel. MDA-MB-231 and BT549 cells at a density of 1.25×10^5^ cells/ml were planted on the upper surface. Migrating and invading cells were fixed with 4% PFA before 0.5% crystal violet staining. The remaining cells were recorded photographically through Zeiss microscope Axio Lab A1 and counted in different fields triply.

### Xenograft Tumor Growth Assays

2×10^6^ MDA-MB-231 cells were subcutaneously inoculated into the dorsal flanks of nude mice. After modeling, nude mice were divided into the control group, low dose group (SA, 25mg/kg/d), and high dose group (SA, 50mg/kg/d) randomly (n=5). SA were administered to mice through intraperitoneal injection. After the mice were euthanized, tumor weights and volumes were measured. All animal experiments were performed according to the institutional guidelines of Sichuan University and Chengdu University of Traditional Chinese Medicine.

### Immunohistochemistry

Then, we used IHC staining to visualize specific protein expression in tissue sections. Tumors were collected after the scarification of nude mice at the end point of the animal study. The cut sections were deparaffinized and rehydrated. Sodium citrate 10 mM, pH 6.0 was used for antigen retrieval at a high temperature. Then, we blocked the section with 5% goat serum for 1h at room temperature. The primary antibodies against PD-L1 were probed with slides at 4°C overnight. After rinsing with TBST three times, the slides were incubated with HRP secondary conjugates for at least 30min at room temperature. SignalStain DAB Substrate Kit was used to detect PD-L1 expression in tumor samples. The nuclei were stained with hematoxylin. The stained cells were recorded photographically through Zeiss microscope Axio Lab A1.

### Quantitative Real-Time PCR

We used the RNAiso Plus reagent to extract total RNA. We used the PrimeScript RT Reagent Kit with gDNA Eraser to perform reverse-transcription of mRNA. We used the ExiLENT SYBR Green master mix to conduct real-time PCR experiments. GAPDH was selected as the loading control in real-time PCR experiments for mRNA expression analyses. MiRNA was extracted with the miRNeasy Mini Kit from Qiagen (DE). We used the TaqMan MicroRNA Reverse Transcription Kit from Ambion (Life Technologies, USA) to perform the reverse-transcription of miRNA. We used the ExiLENT SYBR Green master mix to conduct real-time PCR experiments. U6 was selected as the loading control in real-time PCR experiments for miRNA expression analyses. The corresponding primers for detecting CD247 (PD-L1 gene), GAPDH, and U6 are listed in the **Table S1**.

### PCR Array

MiRNA extraction, reverse transcription, and real-time PCR reactions were performed as previously described. PCR arrays were performed through miRNA miRNome PCR Panel (Qiagen, DE) according to the manufacturer’s instruction.

### Luciferase Reporter Assay

The pMIR-REPOR miRNA Expression Reporter Vector System was used. Dual-Glo Luciferase Assay System was purchased from Promega (Madison, WI) to evaluate the luciferase activity. We inserted the miR-200c-binding sequence of PD-L1 (with or without mutation) to mimic the regulation of PD-L1 by miR-200c through its 3′UTR. The corresponding reagents were added, and the Renilla luciferase activity and firefly luciferase activity were recorded by SpectraMax M5 according to the manufacturer’s instruction. The primers for amplification are shown in **Table S2**.

### Immunofluorescence Assay

MDA-MB-231 and BT549 cells were permeabilized with 0.1% TritonX-100 and incubated with primary antibodies against Vimentin and PD-L1 at 4°C overnight. After rinsing with TBST three times, the slides were incubated with secondary antibodies (Alexa Fluor 488 Conjugate) for at least 1h at room temperature. The photos were obtained *via* Zeiss microscope Axio Lab A1.

### LDH Cytotoxicity Assay

MDA-MB-231 and BT549 cells were seeded on 96 wells at a density of 1×10^5^ cells/ml, and treated with CTL-mediated lysis, or the combination of SA and CTL-mediated lysis, for 24h. LDH Cytotoxicity was detected by LDH Cytotoxicity Assay Kit (Cayman, USA) according to the manufacturer’s instruction. The absorbance of the reacted reagent would be read by an ELISA plate reader at 490nm. The experiments were repeated at least three times.

### Cell Transfection

MirVana miRNA-200c inhibitor and mirVana miRNA inhibitor Negative Control were obtained from Ambion (Life Technologies, USA). Lipofectamine RNAiMAX Transfection Reagent was obtained from Invitrogen (Life Technologies, USA). 1×10^6^ cells/well of MDA-MB-231 and BT549 cells were seeded on the 100mm dish, and miRNA-lipid complexes were added to the cells with 80% coverage of cells on the dish. The cells were incubated at 37°C in the 5% CO_2_ incubator for 24h transfection.

### Data Analysis

GraphPad Prism 7.0 (Graph Pad Software, San Diego, Calif., USA) was used for statistical analyses. All the data were expressed as means ± standard deviations (SD). Two-tailed student’s t test and one-way ANOVA were used to evaluate the statistical significance of Data (*P < 0.05, **P < 0.01).

## Results

### SA Abates the Proliferative Abilities of TNBC Cells and Induces Apoptosis

**Figure 2:**
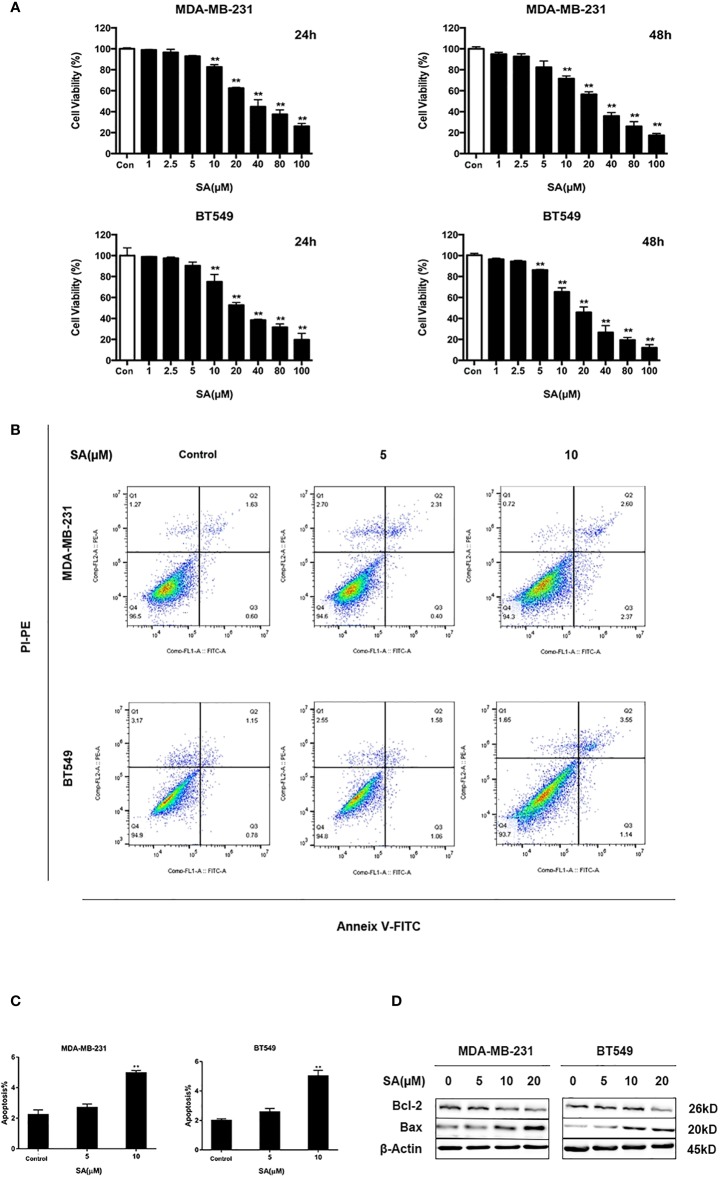
SA inhibits TNBC cells proliferation. **(A)** Cell viability of MDA-MB-231 and BT549 cells after 24h and 48h SA treatment (Compared to the control group, ** p < 0.01). **(B)** Representative images of apoptosis in MDA-MB-231 and BT549 cells determined by Flow cytometry analysis after SA treatment. **(C)** Percentages of apoptotic cells in MDA-MB-231 and BT549 cells after SA treatment (Compared to the control group, ** p < 0.01). **(D)** Western blot analysis of Bax and Bcl-2 after SA treatment (Compared to the control group, ** p < 0.01).

To determine the effect of SA on TNBC cell proliferation, we utilized CCK-8 assay. Results showed that SA (> 10 μM) had a significant inhibitory effect on the cell viability of MDA-MB-231 and BT549 cells after 24h treatment. The IC_50_ values of MDA-MB-231 and BT549 cells were 38.39μM and 27.65μM, respectively (**Figure S1**). SA could inhibit cell proliferation significantly after 48h at the concentration of 5μM (*P* < 0.01). Also, SA treatment showed a dose-dependent inhibition of cell viability in TNBC cells (*P* < 0.01) (**Figure 2A**). Thus, we chose SA for further exploration of cell motility study at 1, 2.5, and 5μM. The results of Annexin V and propidium iodide (PI) staining displayed that SA could increase the percentage of apoptotic cells with the increase of SA concentration, and manifested a significant effect on inducing apoptosis in MDA-MB-231 and BT549 cells after 24h treatment (*P* < 0.01) (**Figures 2B, C**). BCL-2 is an anti-apoptotic regulator and BAX is a pro-apoptotic regulator. Western blot analysis showed that 24h SA treatment significantly down-regulated Bcl-2 expression and up-regulated Bax expression (**Figure 2D**). These results indicate that SA could induce mitochondrial-based apoptosis.

### SA Exerts an Inhibitory Effect on Migration and Invasion of TNBC Cells

**Figure 3:**
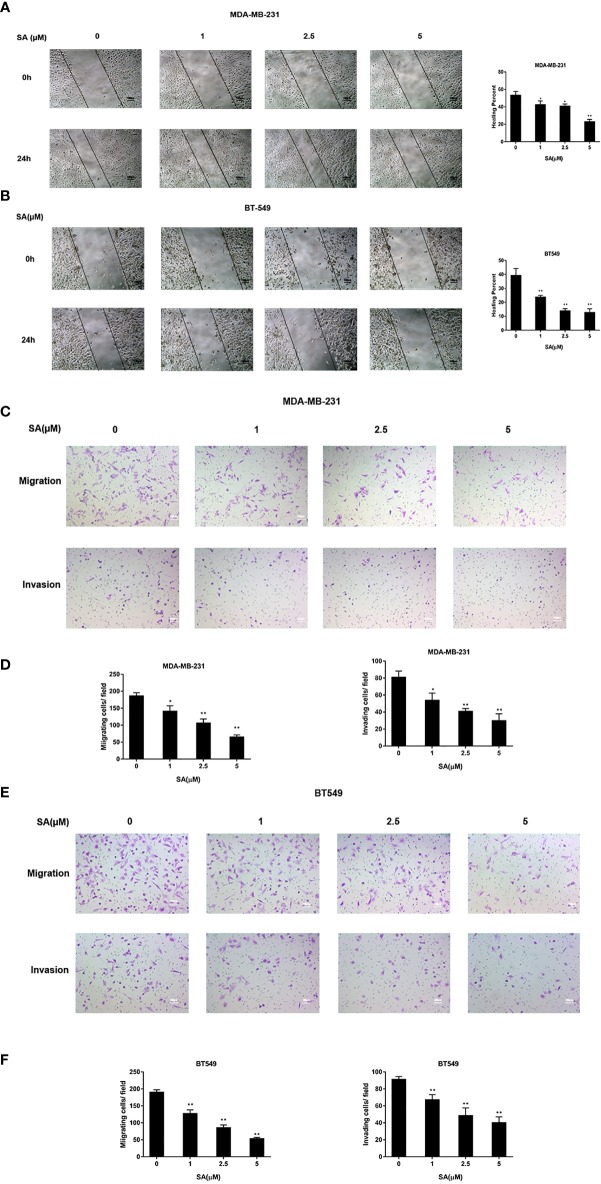
SA attenuates the migratory and invasive abilities of TNBC cells. **(A)** Representative images and percentages of closures of the wound of the wound healing assay in MDA-MB-231 cells after 24h SA treatment. **(B)** Representative images and percentages of healing percent of wound healing assay in BT549 cells after 24h SA treatment. **(C)** Representative images of chamber migration and invasion assay in MDA-MB-231. **(D)** Percentages of migrating and invading in MDA-MB-231 cells after 24h SA treatment (Compared to the control group, * p < 0.05, ** p < 0.01). **(E)** Representative images of chamber migration and invasion assay in BT549 cells. **(F)** Percentages of migrating and invading in BT549 cells after 24h SA treatment (Compared to the control group, ** p < 0.01).

The effect of SA on the cell movement of TNBC cells was detected by a wound healing assay. Results showed that SA could significantly inhibit closures of the wound in MDA-MB-231 and BT549 cells, especially at the concentration of 5μM after 24h treatment (*P* < 0.01) (**Figure 3A**). The effect of SA on migration and invasion of TNBC cells was determined by chamber migration and chamber invasion assay, respectively. Results displayed that the number of MDA-MB-231 cells moving from the upper layer to the lower layer in chamber was dramatically decreased after SA 24h treatment (*P* < 0.01) (**Figures 3B, C**). Meanwhile, the migrating cells and invading cells of BT549 cells also critically abated after SA treatment (*P* < 0.01) (**Figures 3D, E**). Collectively, SA suppresses the migratory and invasive capacities of MDA-MB-231 and BT549 cells.

### SA Suppresses TNBC Growth and Progression *in Vivo*

**Figure 4:**
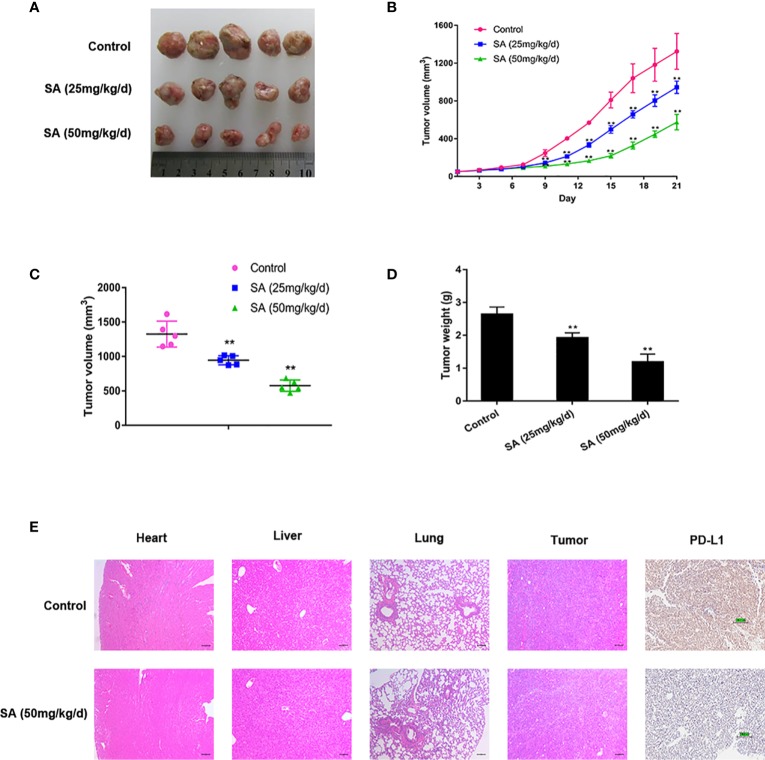
SA inhibits TNBC tumor growth. **(A)**Tumor tissues collected at the end point. **(B)** Tumor volumes in xenograft models during the experiment and measured every three days (Compared to the control group, ** p < 0.01). **(C)** Tumor volumes in xenograft models at the end point (Compared to the control group, ** p < 0.01). **(D)** Tumor weights in xenograft models at the end point (Compared to the control group, ** p < 0.01). **(E)** Representative images of HE staining of normal tissues and IHC analysis of PD-L1 after SA administration.

To determine the anti-cancer effect of SA *in vivo*, SA was given to mice using MDA-MB-231 xenografts by intraperitoneal injection at 25 mg/kg/d (low dose group) and 50 mg/kg/d (high dose group) for 21 days. The tumor bulks were weighed. The tumor volumes were calculated according to a standard formula: (mm3) =L ×W ^2^/2. Results demonstrated that SA could inhibit TNBC tumor volumes significantly, compared to the control group (*P* < 0.01) (**Figures 4A–C**). Additionally, tumor weights decreased dramatically after SA administration (*P* < 0.01) (**Figure 4D**). HE staining exerted that SA had little influence on the micro-morphology of the heart, liver, and lungs even in the high dose group. IHC staining showed that SA treatment could inhibit PD-L1 expression in the high dose group, indicating that SA not only inhibits PD-L1 *in vitro* but also *in vivo* (**Figure 4E**). Altogether, SA inhibits TNBC tumorigenesis *in vivo*.

### SA Increases Lowly Expressed miR-200c in TNBC Cells

**Figure 5:**
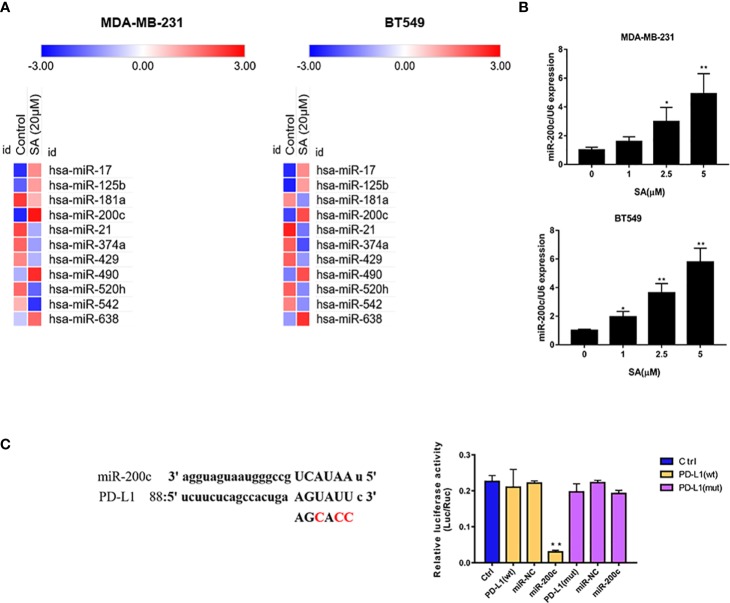
SA increases miR-200c in TNBC cells. **(A)** PCR arrays screened the most significant variation of miRNAs expression in TNBC cells after SA treatment. **(B)** Real-time PCR analysis of miR-200c in MDA-MB-231 and BT549 cells after SA treatment. **(C)** Dual luciferase reporter assay confirmed the direct binding of miR-200c to 3′UTR of PD-L1 in 293 T cells (Compared to the control group, ** p < 0.01).

PCR arrays were utilized to screen the varied miRNAs expression at least 1.5-fold change after 24h 5μM SA treatment in MDA-MB-231 and BT549 cells. Results showed that miR-200c was expressed at a low level in TNBC cells and SA could increase miR-200c in both MDA-MB-231 and BT549 cells, most obviously (**Figure 5A**). Results from a real-time PCR analysis confirmed that SA could significantly increase miR-200c expression in a dose-dependent manner (*P* < 0.01) (**Figure 5B**). Data from PhenomiR 2.0 also displayed that miR-200c was down-regulated in TNBC cell lines and relatively up-regulated in normal breast epithelium (**Figure S2**). MicroRNA.org predicted that miR-200c could target 3′UTR of PD-L1 gene with a relatively high prediction score (**Figure S3**). Then, we used the luciferase activity assay to explore the interactions of miR-200c and PD-L1 gene, and results confirmed the direct binding of miR-200c and the 3′UTR of PD-L1 gene (**Figure 5C**). Taken together, SA could increase miR-200c and miR-200c targets PD-L1.

### High Expression of PD-L1 was Detected in EMT-Activated TNBC Cells

**Figure 6:**
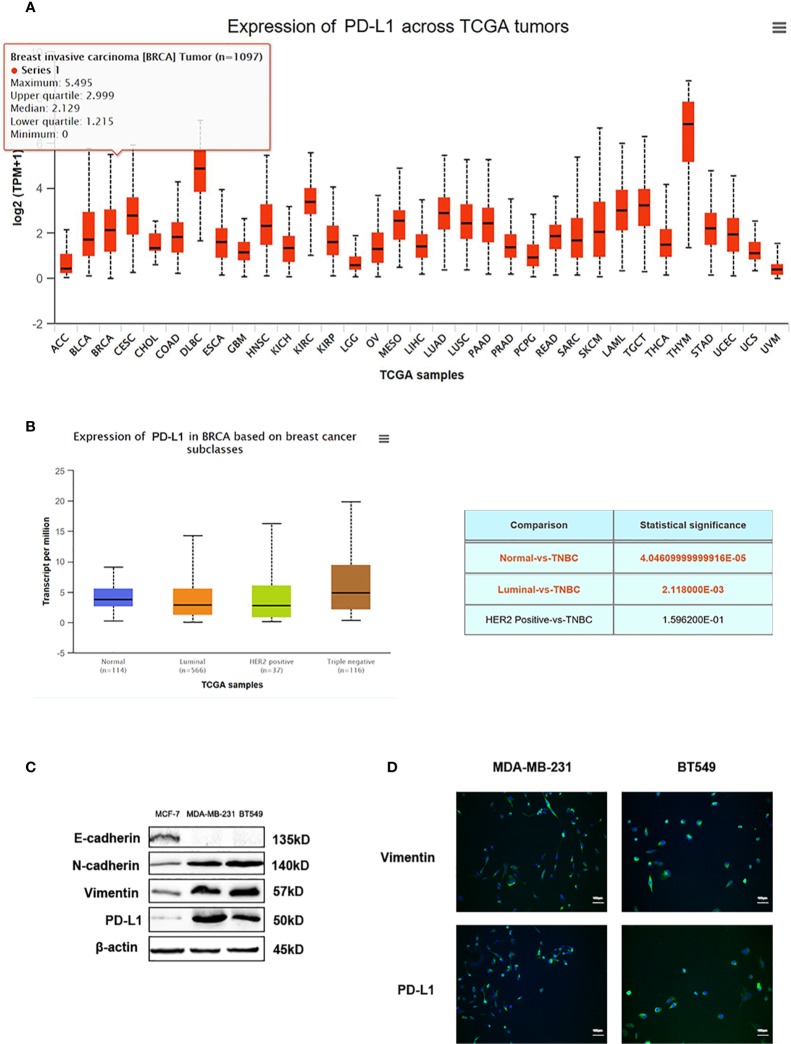
PD-L1 is highly expressed in TNBC. **(A)** The expression of PD-L1 across TGCA tumors. **(B)** The expression of PD-L1 in breast cancer subtypes from TGCA database. **(C)** Western blot analysis of E-cadherin, N-cadherin, Vimentin, and PD-L1 in MDA-MB-231 and BT549 cells. **(D)** Immunofluorescence assay analysis of Vimentin and PD-L1 in MDA-MB-231 and BT549 cells.

Data from TGCA database showed PD-L1 expression in different types of tumors (**Figure 6A**). PD-L1 was relatively highly expressed in TNBC patients among different types of breast cancer, according to the data from TCGA database (**Figure 6B**). Recent reports exert that these EMT-activated TNBC cells are more resistant to immune attacks. Thus, we used western blot analysis and immunofluorescence assay to detect the expression of PD-L1 in EMT-activated TNBC cells. High expression of N-cadherin and Vimentin, and low expression of E-cadherin are considered as tumor markers to identify the EMT process. Results showed that PD-L1 was lowly expressed in MCF-7 cells and EMT-inactivated breast cancer cells, and abundantly expressed in MDA-MB-231 and BT549 cells, EMT-activated TNBC cells (**Figures 6C, D**), suggesting that EMT-activated TNBC cells may be associated with increased PD-L1 expression.

### SA Inhibits PD-L1 in EMT-Activated TNBC Cells Through Up-Regulation of miR-200c

**Figure 7:**
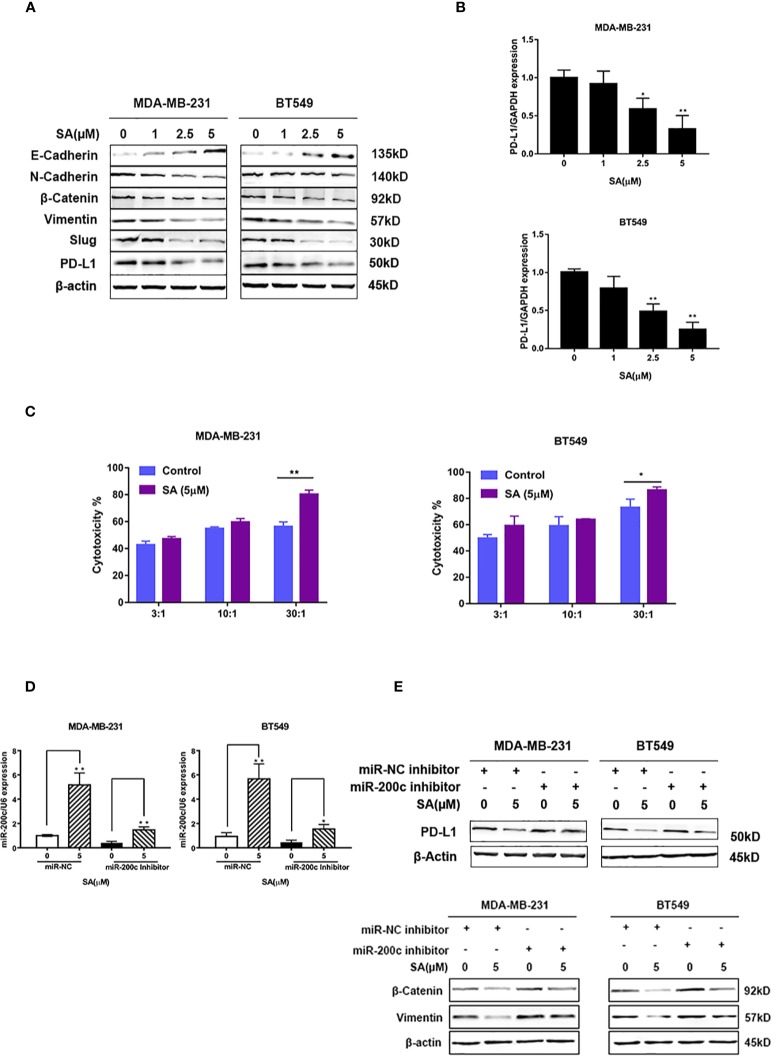
SA inhibits PD-L1 through the up-regulation of miR-200c. **(A)** Western blot analysis of EMT markers and PD-L1 after SA treatment. **(B)** Real-time PCR analysis of PD-L1 in MDA-MB-231 and BT549 cells after SA treatment (Compared to the control group, * p < 0.05,** p < 0.01). **(C)** LDH cytotoxicity of MDA-MB-231 and BT549 cells with or without SA treatment at different effector to target (E: T) ratios (Compared to the control group, * p < 0.05,** p < 0.01). **(D)** Real-time PCR analysis of miR-200c after SA treatment with miR-200c inhibitor interference. **(E)** Western blot analysis of PD-L1, vimentin, and β-catenin after SA treatment with miR-200c inhibitor interference.

Western blot results showed that SA could promote E-cadherin. Additionally, SA could decrease N-cadherin and Vimentin. Also, SA could inhibit the oncogenic transcription regulators in EMT, namely, Snail and Slug. Moreover, SA could decrease PD-L1 expression (**Figure 7A**), suggesting that SA could inhibit EMT process and PD-L1 at the same time. Results from real-time PCR analysis showed that SA could decrease PD-L1 mRNA levels significantly (*P* < 0.01) (**Figure 7B**). LDH cytotoxicity assay displayed that 5μM SA could increase the CTL-mediated killing in MDA-MB-231 and BT549 cells, significantly (*P* < 0.01 and *P* < 0.05, respectively) (**Figure 7C**). Real-time PCR analysis results showed the successful transfection with miR-200c inhibitor and miR-200c negative control in TNBC cells (**Figure 7D**). Western blot results exerted that SA could inhibit PD-L1, and the down-regulation of miR-200c would at least partly reverse the effect of SA. Also, SA could inhibit mesenchymal markers, and the effect would at least partly be reversed after miR-200c inhibitor transfection, suggesting that SA may inhibit EMT through the up-regulation of miR-200c. (**Figure 7E**). Taken together, SA suppresses PD-L1 in EMT-activated TNBC cells *via* the dramatic increase of miR-200c.

## Discussion

It is interesting to find natural compounds with anti-cancer effects from Chinese herbs. According to TCM theory, the initiation of cancer is related to poor physical condition or adverse pathogenic factors (Xu et al., 2006). Thus, improving physical condition and removing pathogenic factors are major aspects used in TCM for cancer treatment. Many natural compounds, such as berberine, curcumin, and resveratrol, isolated from Chinese herbs, exhibited potent anti-cancer activities in preventing tumorigenesis, suppressing metastasis, and controlling tumor growth (Peng et al., 2019a). We found that SA isolated from *Spatholobus suberectus* could inhibit breast cancer proliferation, migration, invasion, and even tumor growth (**Figures 2**–**4**), suggesting that, with further study, SA could be considered with the structure modification to improve the anti-cancer effect.

MiR-200c was lowly expressed in breast cancer and extremely lowly expressed in TNBC cells with the promoter hypermethylation (Vrba et al., 2010) (Sun et al., 2015). A recent study showed that miR-200c could inhibit EMT in breast cancer through the increase of E-cadherin (Tryndyak et al., 2010), and WAS Protein Family Member 3 (WASF3) could inhibit miR-200c expression (Teng et al., 2014). A recent study showed that miR-200c could directly target the PD-L1 gene in lung cancer (Chen et al., 2014). Namely, miR-200c could inhibit both EMT process and PD-L1. In our study, it was the first time in which screen varied miRNAs after SA treatment in TNBC cells, and we found that SA could increase miR-200c expression significantly (**Figures 5A, B**). These data suggested that SA could regulate miR-200c with a potential to affect EMT process and PD-L1 expression.

EMT could increase the migratory and invasive capacity of cancer cells (Demirkan, 2013). The decrease of E-cadherin would promote tumor metastasis (Beavon, 2000). N-cadherin is commonly expressed at a high level in cancer cells and promotes migration (Ramis-Conde et al., 2009). High expression of Vimentin was used to identify the EMT process. Snail and Slug could repress the expression of E-cadherin as the transcription factor. Thus, we utilized western blot to detect the expression of E-cadherin, N-cadherin, Vimentin, Snail, and Slug after SA treatment as the indicator of the EMT process. The results showed that SA could inhibit both the EMT process and PD-L1 expression (**Figure 7A**). PD-L1 is the ligand of PD-1. PD-1-PD-L1 pathway in cancer immunotherapy affect the activity of T-cells (Boussiotis, 2016). Recent studies exerted that anti–PD-1 or anti-PD-L1 antitumor immunity in combination with other therapies could increase efficacy (Mathios et al., 2016). Also, PD-L1 acts as a predictive biomarker for response to cytokine-induced killer cell-based immunotherapy in breast cancer patients, and targeting PD-L1 displayed an anti-cancer effect in the treatment of breast cancer (Dirix et al., 2018; Zhou et al., 2019) Thus, it is interesting to find agents inhibiting PD-L1 expression. Further study exerted that SA could inhibit PD-L1 through the up-regulation of miR-200c (**Figure 7E**). Hence, our study for the first time indicated that SA could inhibit the EMT process, leading to the inhibition of TNBC cell migration and invasion, and could suppress PD-L1 in EMT-activated TNBC cells by increasing miR-200c.

## Conclusion

EMT-activated TNBC cells with high PD-L1 expression are hard to treat. Our study found that SA could inhibit TNBC proliferation, migration, invasion, and growth as a novel drug candidate. We also detect that SA could increase miR-200c expression significantly. Additionally, SA inhibited the EMT process and PD-L1 expression in TNBC cells. SA could increase the cytotoxicity of CTL lysis on TNBC cells. Specifically, SA could inhibit PD-L1 expression through the up-regulation of miR-200c. These data provide novel insights into the correlation of the EMT process and PD-L1 in TNBC and partly provide evidence for the anti-breast cancer effect of *Spatholobus suberectus* Dunn in TNBC therapy.

## Data Availability Statement

Datasets generated for this manuscript can be found in [TCGA] using the accession number TCGA-BRCA. The data are analyzed through online platform UALCAN (http://ualcan.path.uab.edu/analysis.html).

## Ethics Statement

The animal study was reviewed and approved by the Ethics Committee of Chengdu University of Traditional Chinese Medicine.

## Author Contributions

The study was designed by FP, LX, and CP. The manuscript was written by FP and LX. FP performed experiments and analyzed data. CP supported the study and revised the manuscript.

## Funding

The study was supported by the Fundamental Research Funds for the Central Universities (no.YJ201880), and National Natural Science Foundation of China (no.81630101; no.81891012; no.81573663) the Key Project of Science and Technology Department of Sichuan Province (no. 20ZDYF3092), and the Open Research Fund of Chengdu University of Traditional Chinese Medicine Key Laboratory of Systematic Research of Distinctive Chinese Medicine Resources in Southwest China.

## Conflict of Interest

The authors declare that the research was conducted in the absence of any commercial or financial relationships that could be construed as a potential conflict of interest.

## References

[B1] Barrett M. T. Lenkiewicz E. Malasi S. Basu A. Yearley J. H. Annamalai L. (2018). The association of genomic lesions and PD-1/PD-L1 expression in resected triple-negative breast cancers. Breast Cancer Res. 20, 15. 10.1186/s13058-018-1004-0 29996881 PMC6042255

[B2] Beavon I. R. (2000). The E-cadherin-catenin complex in tumour metastasis: structure, function and regulation. Eur. J. Cancer 36 (13 Spec No), 1607–1620. 10.1016/S0959-8049(00)00158-1 10959047

[B3] Bonde M. R. Millar R. L. Ingham J. L. (1973). Induction and identification of sativan and vestitol as two phytoalexins from Lotus corniculatus. Phytochemistry 12 (12), 2957–2959. 10.1016/0031-9422(73)80514-X

[B4] Boussiotis V. A. (2016). Molecular and Biochemical Aspects of the PD-1 Checkpoint Pathway. New Engl. J. Med. 375 (18), 1767–1778. 10.1056/NEJMra1514296 27806234 PMC5575761

[B5] Carey L. A. Perou C. M. Livasy C. A. Dressler L. G. Cowan D. Conway K. (2006). Race, breast cancer subtypes, and survival in the Carolina Breast Cancer Study. JAMA 295 (21), 2492–2502. 10.1001/jama.295.21.2492 16757721

[B6] Chen L. M. Gibbons D. L. Goswami S. Cortez M. A. Ahn Y. H. Byers L. A. (2014). Metastasis is regulated via microRNA-200/ZEB1 axis control of tumour cell PD-L1 expression and intratumoral immunosuppression. Nat. Commun. 5, 12. 10.1038/ncomms6241 PMC4212319 25348003

[B7] Demirkan B. (2013). The Roles of Epithelial-to-Mesenchymal Transition (EMT) and Mesenchymal-to-Epithelial Transition (MET) in Breast Cancer Bone Metastasis: Potential Targets for Prevention and Treatment. J. Clin. Med. 2 (4), 264–282. 10.3390/jcm2040264 26237148 PMC4470149

[B8] Dill E. A. Gru A. A. Atkins K. A. Friedman L. A. Moore M. E. Bullock T. N. (2017). PD-L1 Expression and Intratumoral Heterogeneity Across Breast Cancer Subtypes and Stages. Am. J. Surg. Pathol. 41 (3), 334–342. 10.1097/PAS.0000000000000780 28195880

[B9] Dirix L. Y. Takacs I. Jerusalem G. Nikolinakos P. Arkenau H. T. Forero-Torres A. (2018). Avelumab, an anti-PD-L1 antibody, in patients with locally advanced or metastatic breast cancer: a phase 1b JAVELIN Solid Tumor study. Breast Cancer Res. Tr. 167 (3), 671–686. 10.1007/s10549-017-4537-5 PMC5807460 29063313

[B10] Drasin D. J. Robin T. P. Ford H. L. (2011). Breast cancer epithelial-to-mesenchymal transition: examining the functional consequences of plasticity. Breast Cancer Res. 13 (6), 226. 10.1186/bcr3037 22078097 PMC3326549

[B11] Gralow J. R. Burstein H. J. Wood W. Hortobagyi G. N. Gianni L. von Minckwitz G. (2008). Preoperative therapy in invasive breast cancer: pathologic assessment and systemic therapy issues in operable disease. J. Clin. Oncol. 26 (5), 814–819. 10.1200/JCO.2007.15.3510 18258991

[B12] Ha H. Shim K. S. An H. Kim T. Ma J. Y. (2013). Water extract of Spatholobus suberectus inhibits osteoclast differentiation and bone resorption. BMC Complem. Altern. M. 13, 9. 10.1186/1472-6882-13-112 PMC3664574 23692684

[B13] Hino R. Kabashima K. Kato Y. Yagi H. Nakamura M. Honjo T. (2010). Tumor Cell Expression of Programmed Cell Death-1 Ligand 1 Is a Prognostic Factor for Malignant Melanoma. Cancer 116 (7), 1757–1766. 10.1002/cncr.24899 20143437

[B14] Huang Y. W. Chen L. Feng L. Guo F. J. Li Y. M. (2013). Characterization of Total Phenolic Constituents from the Stems of Spatholobus suberectus Using LC-DAD-MSn and Their Inhibitory Effect on Human Neutrophil Elastase Activity. Molecules 18 (7), 7549–7556. 10.3390/molecules18077549 23807579 PMC6269884

[B15] Ingham J. L. (1978). Isoflavonoid and stilbene phytoalexins of the genus Trifolium. Biochem. Syst. Ecol. 6 (3), 217–223. 10.1016/0305-1978(78)90010-8

[B16] Iorio M. V. Ferracin M. Liu C. G. Veronese A. Spizzo R. Sabbioni S. (2005). MicroRNA gene expression deregulation in human breast cancer. Cancer Res. 65 (16), 7065–7070. 10.1158/0008-5472.CAN-05-1783 16103053

[B17] Li J. Chen L. J. Xiong Y. Q. Zheng X. Xie Q. Q. Zhou Q. (2017). Knockdown of PD-L1 in Human Gastric Cancer Cells Inhibits Tumor Progression and Improves the Cytotoxic Sensitivity to CIK Therapy. Cell Physiol. BiochemI 41 (3), 907–920. 10.1159/00460504 28222426

[B18] Mak M. P. Tong P. Diao L. X. Cardnell R. J. Gibbons D. L. William W. N. (2016). A Patient-Derived, Pan-Cancer EMT Signature Identifies Global Molecular Alterations and Immune Target Enrichment Following Epithelial-to-Mesenchymal Transition. Clin. Cancer Res. 22 (3), 609–620. 10.1158/1078-0432.Ccr-15-0876 26420858 PMC4737991

[B19] Mathios D. Kim J. E. Mangraviti A. Phallen J. Park C. K. Jackson C. M. (2016). Anti-PD-1 antitumor immunity is enhanced by local and abrogated by systemic chemotherapy in GBM. Sci. Transl. Med. 8 (370), 12. 10.1126/scitranslmed.aag2942 PMC5724383 28003545

[B20] Mittal D. Gubin M. M. Schreiber R. D. Smyth M. J. (2014). New insights into cancer immunoediting and its three component phases elimination, equilibrium and escape. Curr. Opin. Immunol. 27, 16–25. 10.1016/j.coi.2014.01.004 24531241 PMC4388310

[B21] Momtazi A. A. Shahabipour F. Khatibi S. Johnston T. P. Pirro M. Sahebkar A. (2016). Curcumin as a MicroRNA Regulator in Cancer: A Review. Rev. Physiol. Biochem. Pharmacol. 171, 1–38. 10.1007/112_2016_3 27457236

[B22] Okazaki T. Honjo T. (2007). PD-1 and PD-1 ligands: from discovery to clinical application. Int. Immunol. 19 (7), 813–824. 10.1093/intimm/dxm057 17606980

[B23] Peng F. Xie X. F. Peng C. (2019a). Chinese Herbal Medicine-Based Cancer Therapy: Novel Anticancer Agents Targeting MicroRNAs to Regulate Tumor Growth and Metastasis. Am. J. Chin. Med. 47 (8), 1711–1735. 10.1142/s0192415x19500873 31801358

[B24] Peng F. Zhu H. Meng C. W. Ren Y. R. Dai O. Xiong L. (2019b). New Isoflavanes from Spatholobus suberectus and Their Cytotoxicity against Human Breast Cancer Cell Lines. Molecules 24 (18), 8. 10.3390/molecules24183218 PMC6766798 31487934

[B25] Qian B. Nag S. A. Su Y. Voruganti S. Qin J. J. Zhang R. (2013). miRNAs in cancer prevention and treatment and as molecular targets for natural product anticancer agents. Curr. Cancer Drug Targets 13 (5), 519–541. 10.2174/15680096113139990031 23597193

[B26] Ramis-Conde I. Chaplain M. A. Anderson A. R. Drasdo D. (2009). Multi-scale modelling of cancer cell intravasation: the role of cadherins in metastasis. Phys. Biol. 6 (1), 016008. 10.1088/1478-3975/6/1/016008 19321920

[B27] Siegel R. Naishadham D. Jemal A. (2013). Cancer statistics 2013. CA Cancer J. Clin. 63 (1), 11–30. 10.3322/caac.21166 23335087

[B28] Sun Q. Liu T. Yuan Y. Guo Z. Xie G. Du S. (2015). MiR-200c inhibits autophagy and enhances radiosensitivity in breast cancer cells by targeting UBQLN1. Int. J. Cancer 136 (5), 1003–1012. 10.1002/ijc.29065 25044403

[B29] Sun J. Q. Zhang G. L. Zhang Y. Nan N. Sun X. Yu M. W. (2016). Spatholobus suberectus Column Extract Inhibits Estrogen Receptor Positive Breast Cancer via Suppressing ER MAPK PI3K/AKT Pathway. Evid-Based Compl. Alt. (7):1–13. 10.1155/2016/2934340 PMC5209621 28096885

[B30] Takashima Y. Kaneko Y. Kobayashi Y. (2010). Synthetic access to optically active isoflavans by using allylic substitution. Tetrahedron 66 (1), 197–207. 10.1016/j.tet.2009.10.116

[B31] Teng Y. Mei Y. Hawthorn L. Cowell J. K. (2014). WASF3 regulates miR-200 inactivation by ZEB1 through suppression of KISS1 leading to increased invasiveness in breast cancer cells. Oncogene 33 (2), 203–211. 10.1038/onc.2012.565 23318438 PMC3998093

[B32] Tryndyak V. P. Beland F. A. Pogribny I. P. (2010). E-cadherin transcriptional down-regulation by epigenetic and microRNA-200 family alterations is related to mesenchymal and drug-resistant phenotypes in human breast cancer cells. Int. J. Cancer 126 (11), 2575–2583. 10.1002/ijc.24972 19839049

[B33] Vrba L. Jensen T. J. Garbe J. C. Heimark R. L. Cress A. E. Dickinson S. (2010). Role for DNA methylation in the regulation of miR-200c and miR-141 expression in normal and cancer cells. PloS One 5 (1), e8697. 10.1371/journal.pone.0008697 20084174 PMC2805718

[B34] Wang Z. Wang D. Han S. Wang N. Mo F. Loo T. (2013). Bioactivity-guided Identification and Cell Signaling Technology to Delineate the Lactate Dehydrogenase a Inhibition Effects of Spatholobus Suberectus on Breast Cancer. PloS One 8 (2), e56631. 10.1371/journal.pone.0056631 23457597 PMC3572989

[B35] Xu W. Towers A. D. Li P. Collet J. P. (2006). Traditional Chinese medicine in cancer care: perspectives and experiences of patients and professionals in China. Eur. J. Cancer Care 15 (4), 397–403. 10.1111/j.1365-2354.2006.00685.x 16968323

[B36] Zhou Z. Q. Zhao J. J. Pan Q. Z. Chen C. L. Liu Y. Tang Y. (2019). PD-L1 expression is a predictive biomarker for CIK cell-based immunotherapy in postoperative patients with breast cancer. J. Immunother. Cancer 7 (1), 14. 10.1186/s40425-019-0696-8 31455411 PMC6712838

