# Supplementary figures and images for: (-)-Sativan Inhibits Tumor Development and Regulates miR-200c/PD-L1 in Triple Negative Breast Cancer Cells
Source: Front Pharmacol. 2020 Mar 13;11:251. doi: 10.3389/fphar.2020.00251 (PMC7082844; doi:10.3389/fphar.2020.00251)

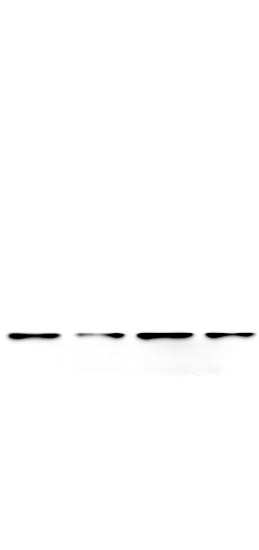

Supplement: Supplementary file 1 [file DataSheet_1.zip › Figure 7E/BT549/Vimentin.tif]

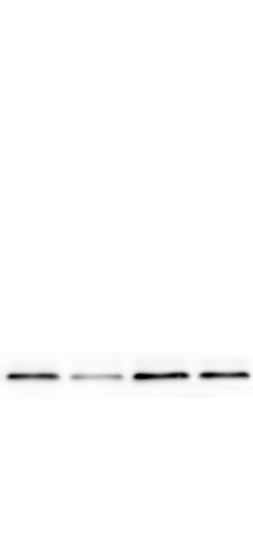

Supplement: Supplementary file 1 [file DataSheet_1.zip › Figure 7E/MDA-MB-231/Vimentin.tif]

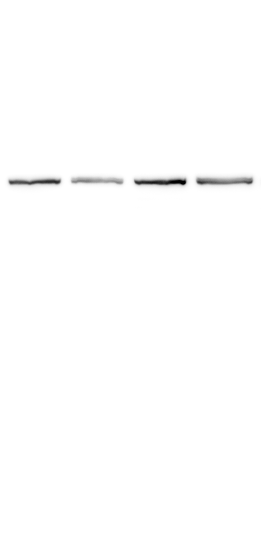

Supplement: Supplementary file 1 [file DataSheet_1.zip › Figure 7E/MDA-MB-231/beta-Catenin.tif]

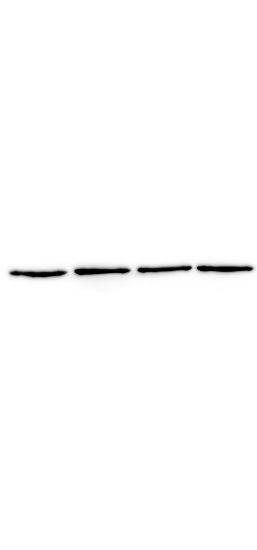

Supplement: Supplementary file 1 [file DataSheet_1.zip › Figure 7E/MDA-MB-231/beta-actin.tif]

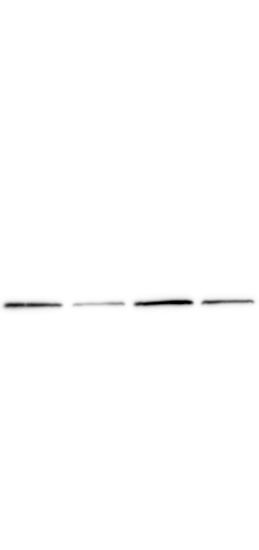

Supplement: Supplementary file 1 [file DataSheet_1.zip › Figure 7E/BT549/beta-Catenin.tif]

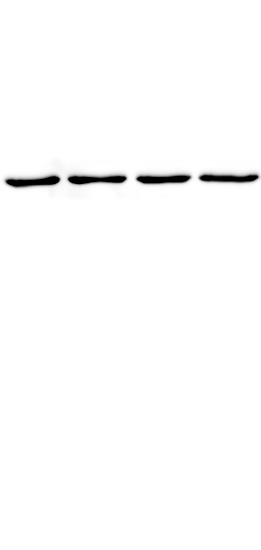

Supplement: Supplementary file 1 [file DataSheet_1.zip › Figure 7E/BT549/beta-actin.tif]

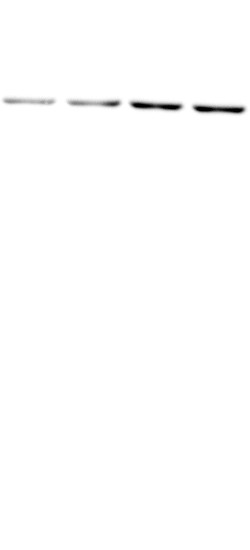

Supplement: Supplementary file 1 [file DataSheet_1.zip › Figure 1D/BT549/BT549 BAX.jpg]

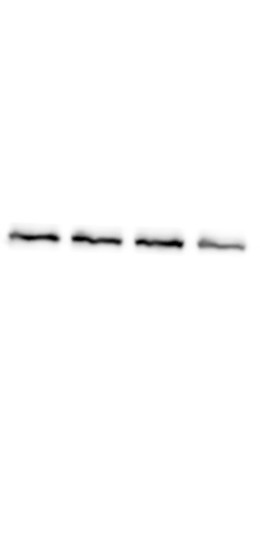

Supplement: Supplementary file 1 [file DataSheet_1.zip › Figure 1D/BT549/BT549 BCL2.jpg]

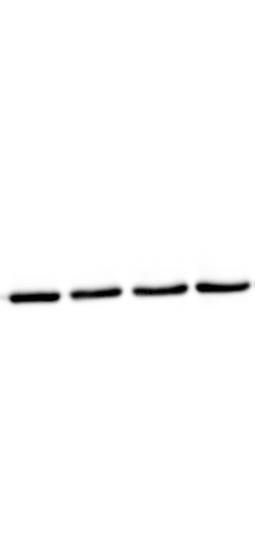

Supplement: Supplementary file 1 [file DataSheet_1.zip › Figure 1D/BT549/BT549 actin.jpg]

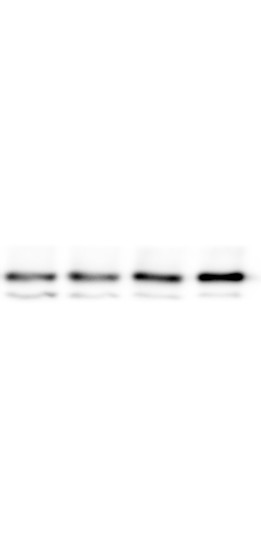

Supplement: Supplementary file 1 [file DataSheet_1.zip › Figure 1D/MDA-MB-231/MDA-MB-231 BAX.jpg]

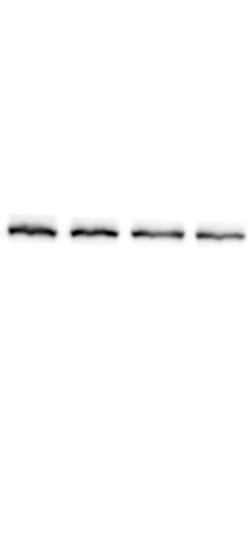

Supplement: Supplementary file 1 [file DataSheet_1.zip › Figure 1D/MDA-MB-231/MDA-MB-231 BCL2.jpg]

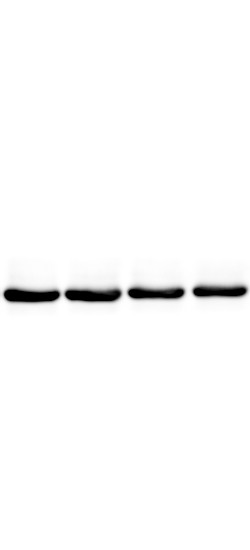

Supplement: Supplementary file 1 [file DataSheet_1.zip › Figure 1D/MDA-MB-231/MDA-MB-231 actin.jpg]

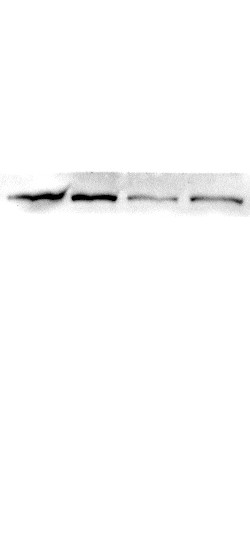

Supplement: Supplementary file 1 [file DataSheet_1.zip › Figure 7A/MDA-MB-231/MDA-MB-231 slug.jpg]

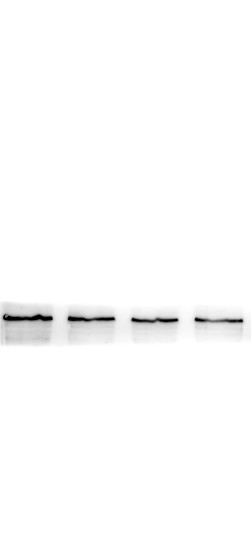

Supplement: Supplementary file 1 [file DataSheet_1.zip › Figure 7A/MDA-MB-231/MDA-MB-231 beta-catenin.jpg]

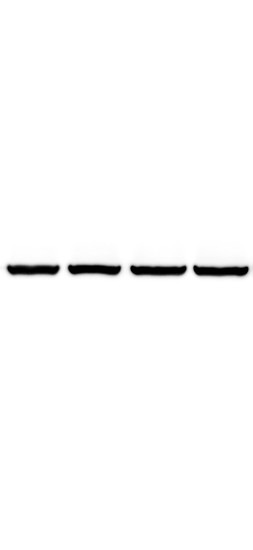

Supplement: Supplementary file 1 [file DataSheet_1.zip › Figure 7A/MDA-MB-231/MDA-MB-231 actin.jpg]

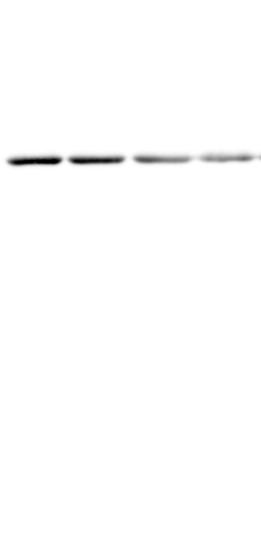

Supplement: Supplementary file 1 [file DataSheet_1.zip › Figure 7A/MDA-MB-231/MDA-MB-231 Vimentin.jpg]

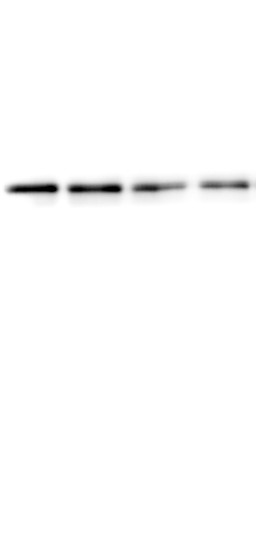

Supplement: Supplementary file 1 [file DataSheet_1.zip › Figure 7A/MDA-MB-231/MDA-MB-231 PD-L1.jpg]

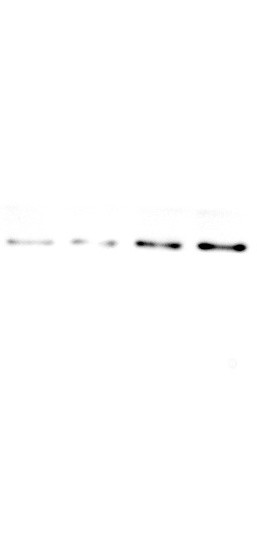

Supplement: Supplementary file 1 [file DataSheet_1.zip › Figure 7A/BT549/BT549 E-cadherin.jpg]

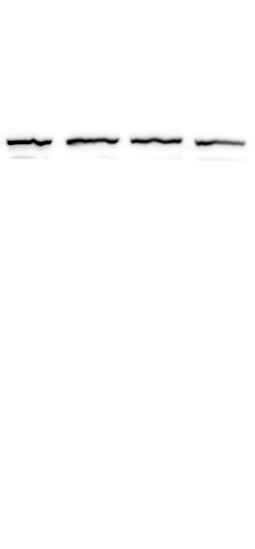

Supplement: Supplementary file 1 [file DataSheet_1.zip › Figure 7A/BT549/BT549 N-cadherin.jpg]

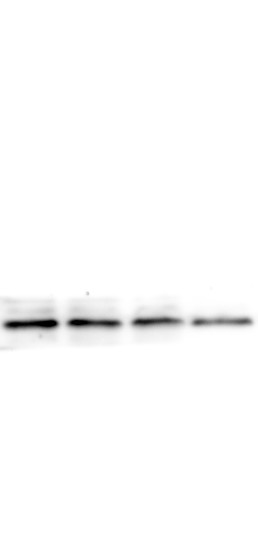

Supplement: Supplementary file 1 [file DataSheet_1.zip › Figure 7A/BT549/BT549 PD-L1.jpg]

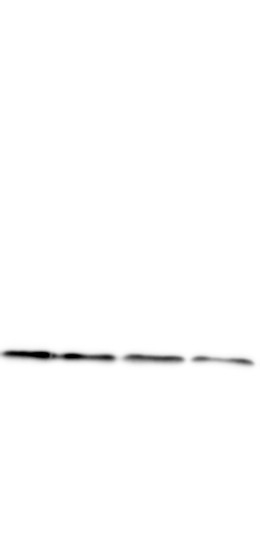

Supplement: Supplementary file 1 [file DataSheet_1.zip › Figure 7A/BT549/BT549 Vimentin.jpg]

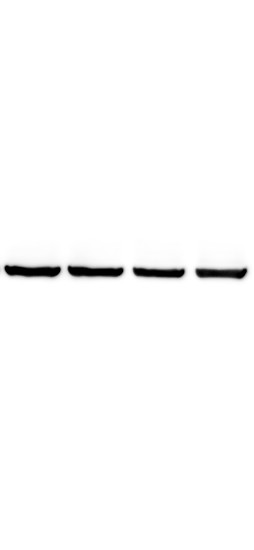

Supplement: Supplementary file 1 [file DataSheet_1.zip › Figure 7A/BT549/BT549 actin.jpg]

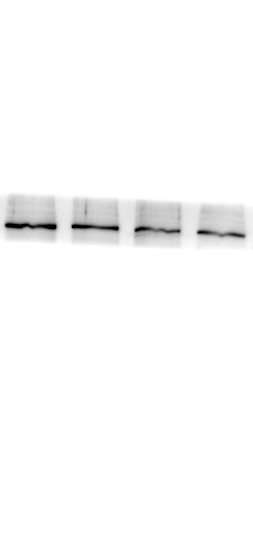

Supplement: Supplementary file 1 [file DataSheet_1.zip › Figure 7A/BT549/BT549 beta-catenin.jpg]

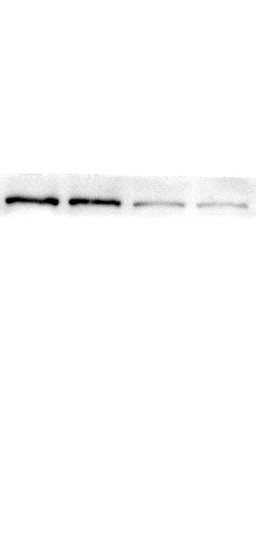

Supplement: Supplementary file 1 [file DataSheet_1.zip › Figure 7A/BT549/BT549 slug.jpg]

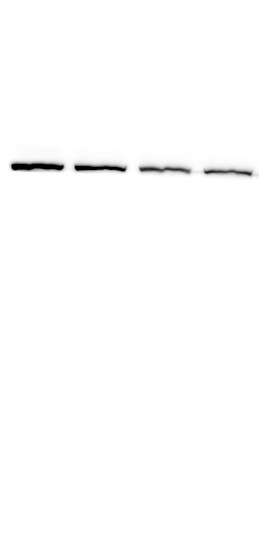

Supplement: Supplementary file 1 [file DataSheet_1.zip › Figure 7A/MDA-MB-231/MDA-MB-231 N-cadherin.jpg]

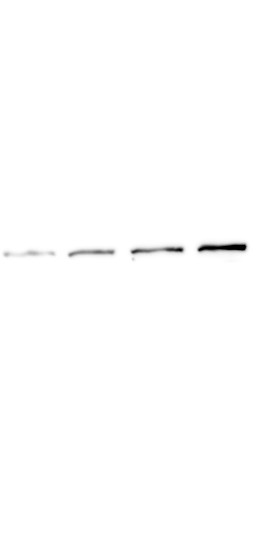

Supplement: Supplementary file 1 [file DataSheet_1.zip › Figure 7A/MDA-MB-231/MDA-MB-231 E-cadherin.jpg]

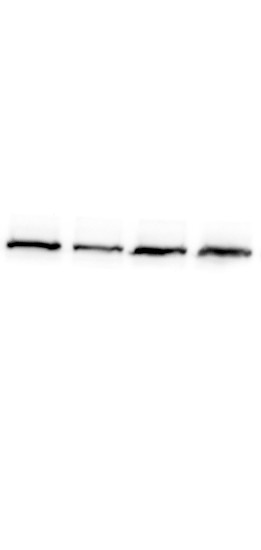

Supplement: Supplementary file 1 [file DataSheet_1.zip › Figure 7E/MDA-MB-231/MDA-MB-231 PD-L1.jpg]

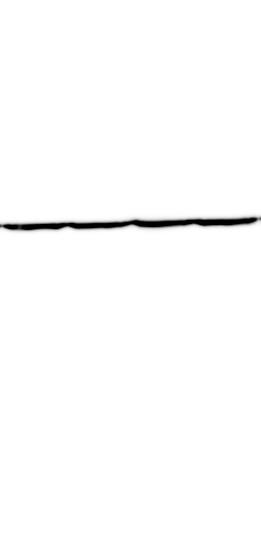

Supplement: Supplementary file 1 [file DataSheet_1.zip › Figure 7E/BT549/BT549 actin.jpg]

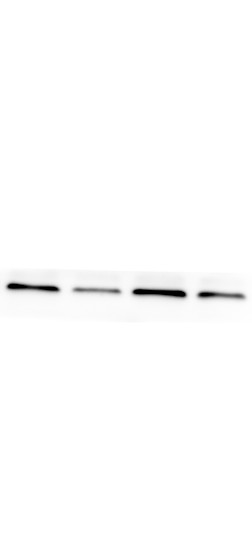

Supplement: Supplementary file 1 [file DataSheet_1.zip › Figure 7E/BT549/BT549 PD-L1.jpg]

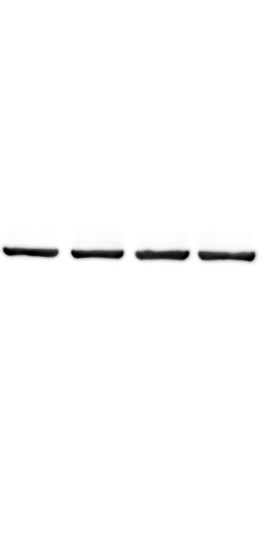

Supplement: Supplementary file 1 [file DataSheet_1.zip › Figure 7E/MDA-MB-231/MDA-MB-231 actin.jpg]

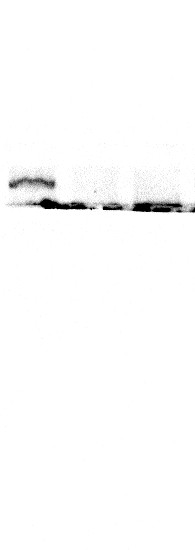

Supplement: Supplementary file 1 [file DataSheet_1.zip › Figure 6C/E-cadherin.jpg]

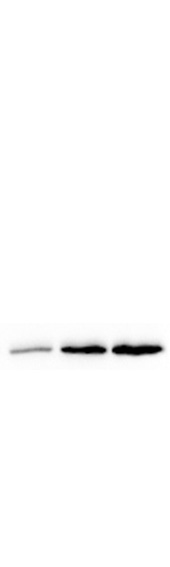

Supplement: Supplementary file 1 [file DataSheet_1.zip › Figure 6C/N-cadherin.jpg]

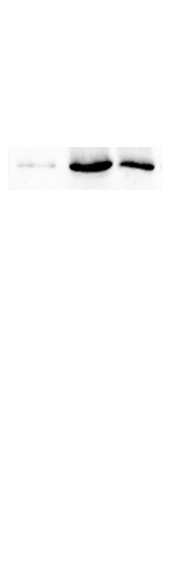

Supplement: Supplementary file 1 [file DataSheet_1.zip › Figure 6C/PD-L1.jpg]

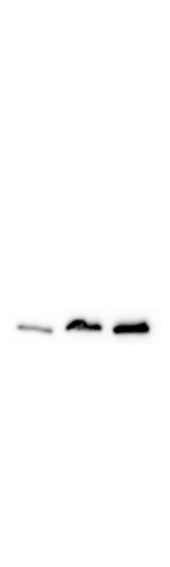

Supplement: Supplementary file 1 [file DataSheet_1.zip › Figure 6C/Vimentin.jpg]

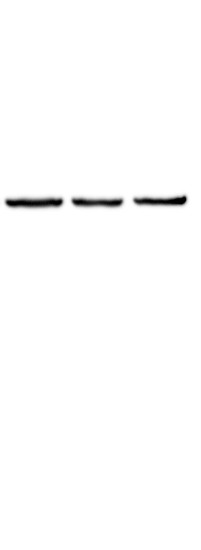

Supplement: Supplementary file 1 [file DataSheet_1.zip › Figure 6C/actin.jpg]
