# Supplementary material for: (-)-Sativan Inhibits Tumor Development and Regulates miR-200c/PD-L1 in Triple Negative Breast Cancer Cells
Source: Front Pharmacol. 2020 Mar 13;11:251. doi: 10.3389/fphar.2020.00251 (PMC7082844; doi:10.3389/fphar.2020.00251)
Supplement: Supplementary file 2 [file DataSheet_2.pdf]

(-)-Sativan inhibits tumor development and regulates miR-200c/PD-L1 in triple negative breast cancer cells

Fu Peng[1,2], Liang Xiong[2], Cheng Peng[2*]

1. *Key Laboratory of Drug-Targeting and Drug Delivery System of the Education Ministry and Sichuan Province, West China School of Pharmacy, Sichuan University, Chengdu, China.*
2. *Chengdu University of Traditional Chinese Medicine, Chengdu, China.*

Correspondence: Cheng Peng, Tel: +86-028-6180-0018, Fax: +86-028-6180-0018, Email: pengchengchengdu@126.com

Table S1. The primers used for Real-time PCR

**CD247**	(F) TGCCGACTACAAGCGAATTACTG
	(R) CTGCTTGTCCAGATGACTTCGG
**GAPDH**	(F) GACTCATGACCACAGTCCATGC
	(R) AGAGGCAGGGATGATGTTCTG
**U6**	(F) CTCGCTTCGGCAGCACA
	(R) AACGCTTCACGAATTTGCGT

Table S2. The primers used for colony PCR

**CD247 wide-type**	(F) CGCGCTCGAGGTTTGGGTCATGCTGGGGGA
	(R) CGCGAAGCTTCTTTCTCCCTGTCACAGGCG
**CD247 mut**	(F) ATTATCACTCTGGTCTATGACATTTGGAGGTCTGCTAATCCAGCATTG GAACTTCTGATC
	(R) CATAGACCAGAGTGATAATAGTATATTAATTTCATGGGAAG

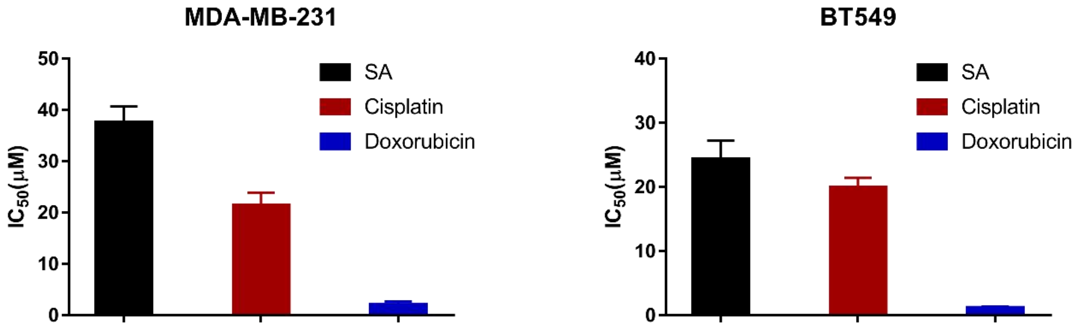

Fig S1. The IC_{50} values of MDA-MB-231 and BT549 cells with the treatment of SA, cisplatin, and doxorubicin for 24 h.

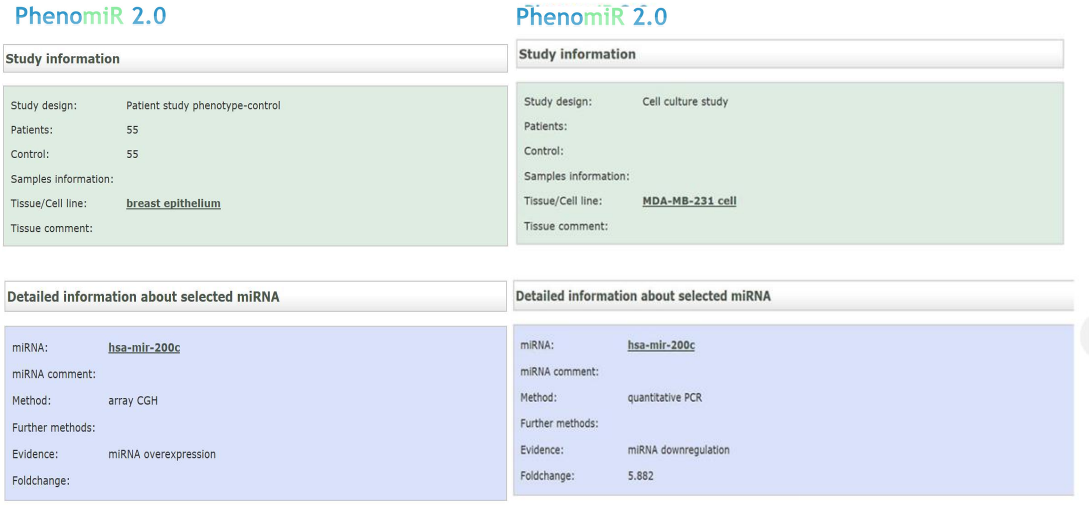

Fig S2. PhenomiR 2.0 (http://mips.helmholtz-muenchen.de/phenomir) showed that miR-200c was highly expressed in normal breast epithelium, and was lowly expressed in TNBC cell line.

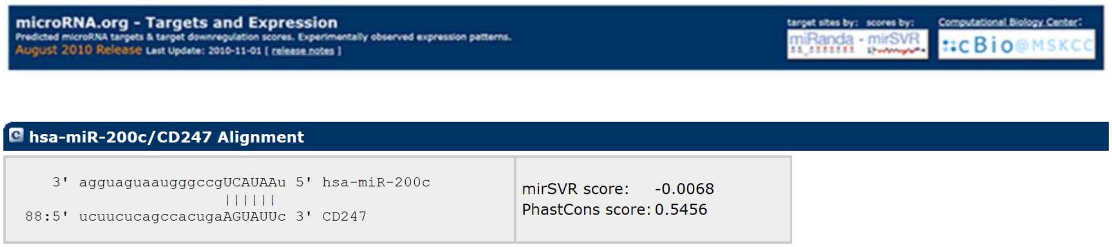

Fig S3. MicroRNA.org (http://www.microrna.org/microrna/getGeneForm.do) predicted that CD247 was one of the most potential targets of miR-200c with the relatively high prediction scores.
